# 8-Hy­droxy­quinolinium 2-carb­oxy­acetate

**DOI:** 10.1107/S1600536810030990

**Published:** 2010-08-11

**Authors:** Ching Kheng Quah, Wan-Sin Loh, Madhukar Hemamalini, Hoong-Kun Fun

**Affiliations:** a X-ray Crystallography Unit, School of Physics, Universiti Sains Malaysia, 11800 USM, Penang, Malaysia

## Abstract

In the title compound, C_9_H_8_NO^+^·C_3_H_3_O_4_^−^, the cation and anion are each essentially planar, with maximum deviations of 0.043 (1) and 0.060 (1) Å, respectively. The dihedral angle between these two planes is 2.20 (4)°. The conformation of the anion is stabilized by an intra­molecular O—H⋯O hydrogen bond, which forms an *S*(6) ring motif. The hy­droxy group of the oxine unit makes a hydrogen bond with the one of the O atoms of the carboxyl­ate group of the 2-carb­oxy­acetate anion. Two other carboxyl­ate O atoms form *R*_2_^2^(7) ring motifs *via* inter­molecular C—H⋯O and N—H⋯O hydrogen bonds. The crystal structure is consolidated by weak inter­molecular C—H⋯O inter­actions, which link the cations and anions into a three-dimensional network.

## Related literature

For background to and the biological activity of oxines, see: Balasubramanian & Muthiah (1996*a* ▶,*b* ▶). For related structures, see: Banerjee *et al.* (1984 ▶); Loh *et al.* (2010 ▶). For the stability of the temperature controller used in the data collection, see: Cosier & Glazer (1986 ▶). For bond-length data, see: Allen *et al.* (1987 ▶). For hydrogen-bond motifs, see: Bernstein *et al.* (1995 ▶).
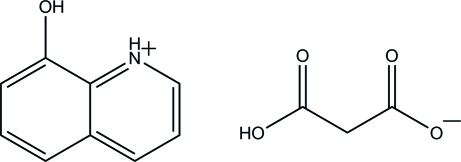


## Experimental

### 

#### Crystal data

C_9_H_8_NO^+^·C_3_H_3_O_4_^−^
*M* *_r_* = 249.22
Monoclinic, 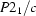

*a* = 8.7089 (4) Å
*b* = 5.2930 (2) Å
*c* = 23.4672 (9) Å
β = 90.999 (3)°
*V* = 1081.58 (8) Å^3^
*Z* = 4
Mo *K*α radiation
μ = 0.12 mm^−1^
*T* = 100 K
0.52 × 0.17 × 0.07 mm

#### Data collection

Bruker SMART APEXII CCD area-detector diffractometer
Absorption correction: multi-scan (*SADABS*; Bruker, 2009 ▶) *T*_min_ = 0.929, *T*_max_ = 0.992
12061 measured reflections
3170 independent reflections
2552 reflections with *I* > 2σ(*I*)
*R*_int_ = 0.041

#### Refinement

*R*[*F* ^2^ > 2σ(*F* ^2^)] = 0.043
*wR*(*F* ^2^) = 0.118
*S* = 1.04
3170 reflections
207 parameters
All H-atom parameters refined
Δρ_max_ = 0.33 e Å^−3^
Δρ_min_ = −0.29 e Å^−3^

### 

Data collection: *APEX2* (Bruker, 2009 ▶); cell refinement: *SAINT* (Bruker, 2009 ▶); data reduction: *SAINT*; program(s) used to solve structure: *SHELXTL* (Sheldrick, 2008 ▶); program(s) used to refine structure: *SHELXTL*; molecular graphics: *SHELXTL*; software used to prepare material for publication: *SHELXTL* and *PLATON* (Spek, 2009 ▶).

## Supplementary Material

Crystal structure: contains datablocks global, I. DOI: 10.1107/S1600536810030990/bt5313sup1.cif

Structure factors: contains datablocks I. DOI: 10.1107/S1600536810030990/bt5313Isup2.hkl

Additional supplementary materials:  crystallographic information; 3D view; checkCIF report

## Figures and Tables

**Table 1:** Hydrogen-bond geometry (Å, °)

*D*—H⋯*A*	*D*—H	H⋯*A*	*D*⋯*A*	*D*—H⋯*A*
O1—H1*O*1⋯O5^i^	0.97 (2)	1.67 (2)	2.6439 (12)	178.1 (14)
O2—H1*O*2⋯O4	0.97 (2)	1.55 (2)	2.4963 (14)	162 (2)
N1—H1*N*1⋯O5^ii^	0.95 (2)	1.74 (2)	2.6809 (14)	170.9 (17)
C2—H2*A*⋯O4^ii^	0.928 (16)	2.373 (17)	3.1735 (15)	144.4 (14)
C2—H2*A*⋯O2^iii^	0.928 (16)	2.423 (16)	3.0663 (15)	126.5 (13)
C6—H6*A*⋯O3^iv^	0.964 (16)	2.462 (16)	3.4202 (16)	172.5 (13)

Symmetry codes: (i) 
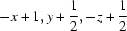
; (ii) 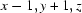; (iii) 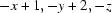; (iv) 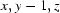.

**Fig. 1.**
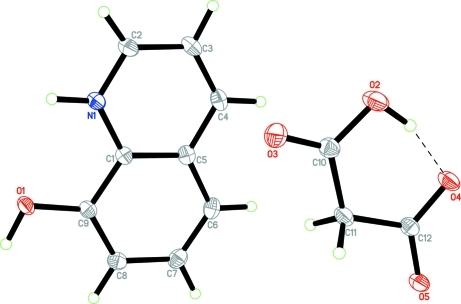
The molecular structure of the title compound showing 50% probability displacement ellipsoids for non-H atoms and the atom-numbering scheme. Intramolecular interaction is shown in dashed line.

**Fig. 2.**
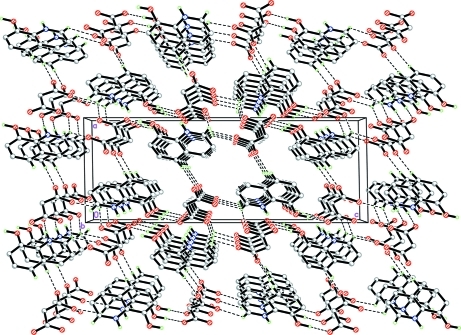
The crystal structure of the title compound viewed along the b axis. H atoms not involved in intermolecular interactions (dashed lines) have been omitted for clarity.

## supplementary crystallographic information

### Comment

Recently, much attention has been devoted to the design and synthesis of supramolecular architectures assembled *via* various weak noncovalent interactions in the crystal structures of oxines (8-hydroxyquinoline), their derivatives and their complexes in a variety of crystalline environments (Balasubramanian & Muthiah, 1996*a*,*b*). Oxine is widely used as analytical reagent. The present study is aimed at understanding the hydrogen-bonding networks in the title compound, (I).

The asymmetric unit of title compound, (Fig. 1), consists of a 8-hydroxyquinolinium cation and a 2-carboxyacetate anion. 8-Hydroxyquinolinium is protonated at atom N1 leading to an enhancement of the internal angle [122.14 (11)°] at C1—N1—C2 compared with neutral quinoline moieties (Banerjee *et al.*, 1984). The 8-hydroxyquinolinium cation and 2-carboxyacetate anion are essentially planar, with a maximum deviation of 0.043 (1) Å for atom C8 and 0.060 (1) Å for atom C11, respectively. The diheral angle between these two planes is 2.20 (4)°, indicating they are approximately parallel to each other. The anion is stabilized by an intramolecular O2–H1O2···O4 hydrogen bond, which forms an S(6) ring motif (Bernstein *et al.*, 1995).

In the solid state (Fig. 2), carboxylate oxygen atoms (O4 and O5) form *R*_2_^2^(7) ring motifs *via* intermolecular C2–H2A···O4 and N1–H1N1···O5 hydrogen bonds. The hydroxy group (O1–H1O1) of the oxine moiety makes a hydrogen bond with the O5 atom of the carboxylate group of the 2-carboxyacetate anion. The crystal structure is consolidated by weak intermolecular C2–H2A···O2 and C6–H6A···O3 interactions. The cations and anions are linked by these interactions into three-dimensional network.

### Experimental

A hot methanol solution (20 ml) of 8-hydroxyquinolinine (29 mg, Merck) and malonic acid (20.8 mg, Acros) was mixed and warmed over a magnetic stirrer hotplate for a few minutes. The resulting solution was allowed to cool slowly at room temperature and crystals of the title compound appeared after a few days.

### Refinement

All H atoms were located in a difference Fourier map and refined freely.

### Crystal data

**Table:**

C_9_H_8_NO^+^·C_3_H_3_O_4_^−^	*F*(000) = 520
*M**_r_* = 249.22	*D*_x_ = 1.530 Mg m^−^^3^
Monoclinic, *P*2_1_/*c*	Mo *K*α radiation, λ = 0.71073 Å
Hall symbol: -P 2ybc	Cell parameters from 3422 reflections
*a* = 8.7089 (4) Å	θ = 3.5–30.0°
*b* = 5.2930 (2) Å	µ = 0.12 mm^−^^1^
*c* = 23.4672 (9) Å	*T* = 100 K
β = 90.999 (3)°	Plate, yellow
*V* = 1081.58 (8) Å^3^	0.52 × 0.17 × 0.07 mm
*Z* = 4	

### Data collection

**Table:**

Bruker SMART APEXII CCD area-detector diffractometer	3170 independent reflections
Radiation source: fine-focus sealed tube	2552 reflections with *I* > 2σ(*I*)
graphite	*R*_int_ = 0.041
φ and ω scans	θ_max_ = 30.1°, θ_min_ = 2.3°
Absorption correction: multi-scan (*SADABS*; Bruker, 2009)	*h* = −11→12
*T*_min_ = 0.929, *T*_max_ = 0.992	*k* = −7→7
12061 measured reflections	*l* = −33→32

### Refinement

**Table:**

Refinement on *F*^2^	Primary atom site location: structure-invariant direct methods
Least-squares matrix: full	Secondary atom site location: difference Fourier map
*R*[*F*^2^ > 2σ(*F*^2^)] = 0.043	Hydrogen site location: inferred from neighbouring sites
*wR*(*F*^2^) = 0.118	All H-atom parameters refined
*S* = 1.04	*w* = 1/[σ^2^(*F*_o_^2^) + (0.0618*P*)^2^ + 0.2285*P*] where *P* = (*F*_o_^2^ + 2*F*_c_^2^)/3
3170 reflections	(Δ/σ)_max_ < 0.001
207 parameters	Δρ_max_ = 0.33 e Å^−^^3^
0 restraints	Δρ_min_ = −0.29 e Å^−^^3^

### Special details

**Table:**

Experimental. The crystal was placed in the cold stream of an Oxford Cyrosystems Cobra open-flow nitrogen cryostat (Cosier & Glazer, 1986) operating at 100.0 (1) K.
Geometry. All e.s.d.'s (except the e.s.d. in the dihedral angle between two l.s. planes) are estimated using the full covariance matrix. The cell e.s.d.'s are taken into account individually in the estimation of e.s.d.'s in distances, angles and torsion angles; correlations between e.s.d.'s in cell parameters are only used when they are defined by crystal symmetry. An approximate (isotropic) treatment of cell e.s.d.'s is used for estimating e.s.d.'s involving l.s. planes.
Refinement. Refinement of *F*^2^ against ALL reflections. The weighted *R*-factor *wR* and goodness of fit *S* are based on *F*^2^, conventional *R*-factors *R* are based on *F*, with *F* set to zero for negative *F*^2^. The threshold expression of *F*^2^ > σ(*F*^2^) is used only for calculating *R*-factors(gt) *etc*. and is not relevant to the choice of reflections for refinement. *R*-factors based on *F*^2^ are statistically about twice as large as those based on *F*, and *R*- factors based on ALL data will be even larger.

### Fractional atomic coordinates and isotropic or equivalent isotropic displacement parameters (Å^2^)

**Table:**

	*x*	*y*	*z*	*U*_iso_*/*U*_eq_	
O1	0.09573 (11)	0.85826 (16)	0.23539 (4)	0.0178 (2)	
N1	0.16598 (13)	0.89411 (19)	0.12400 (4)	0.0152 (2)	
C1	0.23458 (14)	0.7104 (2)	0.15690 (5)	0.0142 (2)	
C2	0.19403 (16)	0.9186 (2)	0.06877 (5)	0.0183 (3)	
C3	0.29489 (16)	0.7560 (3)	0.04150 (5)	0.0204 (3)	
C4	0.36509 (16)	0.5673 (2)	0.07247 (5)	0.0201 (3)	
C5	0.33690 (14)	0.5386 (2)	0.13142 (5)	0.0165 (2)	
C6	0.40592 (15)	0.3468 (2)	0.16573 (6)	0.0193 (3)	
C7	0.37370 (15)	0.3379 (2)	0.22279 (6)	0.0193 (3)	
C8	0.27372 (15)	0.5121 (2)	0.24808 (5)	0.0172 (2)	
C9	0.19971 (14)	0.6941 (2)	0.21562 (5)	0.0144 (2)	
O2	0.79886 (13)	0.74847 (19)	0.03808 (4)	0.0268 (2)	
O3	0.66717 (12)	0.95987 (18)	0.10240 (4)	0.0237 (2)	
O4	0.96190 (11)	0.38081 (17)	0.06705 (4)	0.0208 (2)	
O5	0.97057 (11)	0.25359 (16)	0.15766 (3)	0.0177 (2)	
C10	0.75145 (15)	0.7864 (2)	0.09062 (5)	0.0176 (2)	
C11	0.80638 (16)	0.5972 (2)	0.13524 (5)	0.0195 (3)	
C12	0.92134 (14)	0.3958 (2)	0.11786 (5)	0.0155 (2)	
H1O1	0.073 (2)	0.817 (4)	0.2748 (9)	0.042 (5)*	
H1O2	0.866 (3)	0.602 (4)	0.0417 (9)	0.057 (6)*	
H1N1	0.098 (2)	1.015 (4)	0.1398 (8)	0.037 (5)*	
H2A	0.141 (2)	1.051 (3)	0.0516 (7)	0.022 (4)*	
H3A	0.3100 (19)	0.781 (3)	0.0005 (7)	0.023 (4)*	
H4A	0.437 (2)	0.447 (3)	0.0560 (7)	0.027 (4)*	
H6A	0.4720 (18)	0.226 (3)	0.1476 (7)	0.019 (4)*	
H7A	0.4224 (19)	0.207 (3)	0.2466 (7)	0.024 (4)*	
H8A	0.2547 (19)	0.505 (3)	0.2888 (7)	0.020 (4)*	
H11A	0.716 (2)	0.510 (4)	0.1503 (8)	0.039 (5)*	
H11B	0.852 (2)	0.689 (4)	0.1680 (8)	0.035 (5)*	

### Atomic displacement parameters (Å^2^)

**Table:**

	*U*^11^	*U*^22^	*U*^33^	*U*^12^	*U*^13^	*U*^23^
O1	0.0235 (5)	0.0173 (4)	0.0128 (4)	0.0039 (4)	0.0051 (3)	0.0010 (3)
N1	0.0187 (5)	0.0143 (4)	0.0128 (5)	−0.0006 (4)	0.0020 (4)	−0.0003 (4)
C1	0.0153 (6)	0.0129 (5)	0.0146 (5)	−0.0014 (4)	0.0013 (4)	−0.0008 (4)
C2	0.0229 (7)	0.0186 (6)	0.0135 (5)	−0.0009 (5)	0.0017 (4)	0.0008 (4)
C3	0.0242 (7)	0.0240 (6)	0.0130 (5)	−0.0009 (5)	0.0041 (5)	−0.0016 (5)
C4	0.0201 (7)	0.0213 (6)	0.0191 (6)	0.0009 (5)	0.0047 (5)	−0.0045 (5)
C5	0.0159 (6)	0.0160 (5)	0.0178 (6)	−0.0018 (4)	0.0017 (4)	−0.0016 (4)
C6	0.0170 (6)	0.0161 (5)	0.0248 (6)	0.0015 (5)	0.0016 (5)	−0.0018 (5)
C7	0.0180 (6)	0.0160 (5)	0.0239 (6)	0.0005 (5)	−0.0007 (5)	0.0035 (5)
C8	0.0186 (6)	0.0169 (5)	0.0160 (5)	−0.0024 (5)	0.0007 (4)	0.0019 (4)
C9	0.0161 (6)	0.0136 (5)	0.0137 (5)	−0.0022 (4)	0.0023 (4)	0.0002 (4)
O2	0.0417 (7)	0.0242 (5)	0.0146 (4)	0.0117 (4)	0.0046 (4)	0.0025 (4)
O3	0.0263 (5)	0.0216 (5)	0.0232 (5)	0.0067 (4)	0.0009 (4)	−0.0014 (4)
O4	0.0293 (5)	0.0205 (4)	0.0127 (4)	0.0049 (4)	0.0051 (3)	0.0001 (3)
O5	0.0228 (5)	0.0175 (4)	0.0128 (4)	0.0018 (3)	0.0023 (3)	0.0008 (3)
C10	0.0197 (6)	0.0170 (5)	0.0160 (5)	0.0001 (5)	0.0005 (4)	−0.0009 (4)
C11	0.0241 (7)	0.0211 (6)	0.0132 (5)	0.0055 (5)	0.0040 (5)	0.0008 (4)
C12	0.0178 (6)	0.0149 (5)	0.0139 (5)	−0.0023 (4)	0.0015 (4)	−0.0013 (4)

### Geometric parameters (Å, °)

**Table:**

O1—C9	1.3434 (15)	C6—H6A	0.963 (17)
O1—H1O1	0.97 (2)	C7—C8	1.4066 (18)
N1—C2	1.3295 (15)	C7—H7A	0.981 (17)
N1—C1	1.3722 (15)	C8—C9	1.3811 (17)
N1—H1N1	0.95 (2)	C8—H8A	0.973 (16)
C1—C5	1.4132 (17)	O2—C10	1.3223 (15)
C1—C9	1.4187 (16)	O2—H1O2	0.97 (2)
C2—C3	1.3933 (18)	O3—C10	1.2107 (16)
C2—H2A	0.928 (17)	O4—C12	1.2521 (14)
C3—C4	1.3727 (19)	O5—C12	1.2683 (14)
C3—H3A	0.982 (16)	C10—C11	1.5198 (17)
C4—C5	1.4174 (17)	C11—C12	1.5231 (17)
C4—H4A	0.977 (18)	C11—H11A	0.98 (2)
C5—C6	1.4222 (17)	C11—H11B	0.990 (19)
C6—C7	1.3736 (18)		
			
C9—O1—H1O1	109.3 (12)	C6—C7—C8	121.81 (12)
C2—N1—C1	122.14 (11)	C6—C7—H7A	119.1 (10)
C2—N1—H1N1	116.1 (11)	C8—C7—H7A	119.1 (10)
C1—N1—H1N1	121.7 (11)	C9—C8—C7	120.74 (11)
N1—C1—C5	119.31 (10)	C9—C8—H8A	118.7 (10)
N1—C1—C9	119.40 (10)	C7—C8—H8A	120.5 (10)
C5—C1—C9	121.28 (11)	O1—C9—C8	124.86 (11)
N1—C2—C3	121.04 (12)	O1—C9—C1	116.96 (10)
N1—C2—H2A	113.5 (10)	C8—C9—C1	118.17 (11)
C3—C2—H2A	125.5 (10)	C10—O2—H1O2	103.7 (13)
C4—C3—C2	119.00 (11)	O3—C10—O2	121.90 (12)
C4—C3—H3A	123.3 (10)	O3—C10—C11	121.81 (11)
C2—C3—H3A	117.6 (10)	O2—C10—C11	116.29 (11)
C3—C4—C5	120.77 (12)	C10—C11—C12	118.50 (10)
C3—C4—H4A	123.2 (10)	C10—C11—H11A	108.4 (12)
C5—C4—H4A	116.1 (10)	C12—C11—H11A	107.4 (12)
C1—C5—C4	117.72 (11)	C10—C11—H11B	109.3 (11)
C1—C5—C6	118.88 (11)	C12—C11—H11B	107.1 (11)
C4—C5—C6	123.40 (12)	H11A—C11—H11B	105.5 (16)
C7—C6—C5	119.01 (12)	O4—C12—O5	124.50 (11)
C7—C6—H6A	122.8 (9)	O4—C12—C11	119.80 (11)
C5—C6—H6A	118.2 (9)	O5—C12—C11	115.70 (10)
			
C2—N1—C1—C5	−0.90 (18)	C5—C6—C7—C8	0.37 (19)
C2—N1—C1—C9	−179.97 (11)	C6—C7—C8—C9	2.3 (2)
C1—N1—C2—C3	0.34 (19)	C7—C8—C9—O1	175.89 (11)
N1—C2—C3—C4	0.4 (2)	C7—C8—C9—C1	−3.83 (18)
C2—C3—C4—C5	−0.5 (2)	N1—C1—C9—O1	2.09 (16)
N1—C1—C5—C4	0.71 (17)	C5—C1—C9—O1	−176.97 (11)
C9—C1—C5—C4	179.77 (11)	N1—C1—C9—C8	−178.17 (11)
N1—C1—C5—C6	−179.22 (11)	C5—C1—C9—C8	2.77 (18)
C9—C1—C5—C6	−0.16 (18)	O3—C10—C11—C12	−175.64 (12)
C3—C4—C5—C1	−0.01 (19)	O2—C10—C11—C12	5.39 (18)
C3—C4—C5—C6	179.92 (13)	C10—C11—C12—O4	−3.81 (18)
C1—C5—C6—C7	−1.42 (18)	C10—C11—C12—O5	175.62 (11)
C4—C5—C6—C7	178.66 (12)		

### Hydrogen-bond geometry (Å, °)

**Table:**

*D*—H···*A*	*D*—H	H···*A*	*D*···*A*	*D*—H···*A*
O1—H1O1···O5^i^	0.97 (2)	1.67 (2)	2.6439 (12)	178.1 (14)
O2—H1O2···O4	0.97 (2)	1.55 (2)	2.4963 (14)	162 (2)
N1—H1N1···O5^ii^	0.95 (2)	1.74 (2)	2.6809 (14)	170.9 (17)
C2—H2A···O4^ii^	0.928 (16)	2.373 (17)	3.1735 (15)	144.4 (14)
C2—H2A···O2^iii^	0.928 (16)	2.423 (16)	3.0663 (15)	126.5 (13)
C6—H6A···O3^iv^	0.964 (16)	2.462 (16)	3.4202 (16)	172.5 (13)

Symmetry codes: (i) −*x*+1, *y*+1/2, −*z*+1/2; (ii) *x*−1, *y*+1, *z*; (iii) −*x*+1, −*y*+2, −*z*; (iv) *x*, *y*−1, *z*.
